# Correction: Unemployment during the Great Recession and Large-for-Gestational-Age births

**DOI:** 10.1371/journal.pone.0236969

**Published:** 2020-07-29

**Authors:** The PLOS ONE Staff

## Notice of republication

This article was republished on July 16, 2020, to correct errors throughout the article that were introduced during the typesetting process. The publisher apologizes for the errors. Please download this article again to view the correct version. The originally published, uncorrected article and the republished, corrected articles are provided here for reference.

## Supporting information

S1 File Originally published, uncorrected article. (PDF)

S2 File Republished, corrected article. (PDF)

## References

[1] Oddo VM, Jones-Smith JC (2020) Unemployment during the Great Recession and Large-for-Gestational-Age births. PLoS ONE 15(5): e0233734 10.1371/journal.pone.0233734 32469967 PMC7259553

