# Supplementary material for: Unemployment during the Great Recession and Large-for-Gestational-Age births
Source: PLoS One. 2020 May 29;15(5):e0233734. doi: 10.1371/journal.pone.0233734 (PMC7259553; doi:10.1371/journal.pone.0233734)
Supplement: S1 Table — (DOCX) [file pone.0233734.s001.docx]

| **S1 Table. Marginal Effects for the Relationship Between Unemployment Rate and LGA Births, 2008-2011** | | | |
| --- | --- | --- | --- |
|  | **n** | **Percentage Point (95% CI)^a^** | **p value** |
| Main Model |  |  |  |
| Unemployment Rate^a,b,c^ | 1,715,052 | 0.11 (-0.02, 0.23) | 0.09 |
| Sensitivity Analyses |  |  |  |
| Unemployment Rate, Including Preterm Births ^a,b,c^ | 1,877,359 | 0.06 (-0.07, 0.19) | 0.10 |
| Unemployment Rate, Among Non-Smokers ^a,b,c^ | 1,656,120 | 0.11 (-0.02, 0.24) | 0.09 |
| Unemployment Rate, Excluding LA County ^a,b,c^ | 1,570,751 | 0.11(-0.02, 0.24) | 0.10 |
| Unemployment Rate, Excluding Pacific Islanders and People of Other Races ^a,b,d^ | 1,707,058 | 0.11 (-0.01, 0.24) | 0.07 |
| Unemployment Rate, Excluding Foreclosure ^a,b,e^ | 1,715,052 | 0.09 (-0.03, 0.22) | 0.15 |
| Unemployment Rate, Pre-Recession Years ^a,b,c,f^ | 2,145,755 | -0.02 (-0.12, -0.09) | 0.78 |
| Secondary Outcomes |  |  |  |
| Very LGA ^a,b,c^ | 1,715,052 | 0.08 (0.03, 0.14) | 0.01 |
| Excess GWG ^a,b,c^ | 1,587,721 | 0.16 (-0.33, 0.66) | 0.52 |
| High Birthweight ^a,b,c^ | 1,715,052 | 0.03 (-0.10, 0.16) | 0.61 |
| Preterm^b,c,g^ | 1,878,201 | -0.13 (-0.25, -0.17) | 0.03 |

CI = confidence interval, GWG = gestational weight gain; LGA= large-for-gestational-age

^a^ Coefficients are estimated for singleton, term births between using logit regression models, with county fixed-effects. The coefficients are multiplied by 100 and can be interpreted as a percentage point.

^b^ County-level unemployment is lagged and reflects the unemployment rate in the year prior to birth.

^c^ Models include an indicator variable for year and control for county-level foreclosure rates, maternal age, parity, race/ethnicity, education, and child gender.

^d^ Model includes an indicator variable for year and controls for county-level foreclosure rates, maternal age, parity, education, and child gender.

^e^ Model includes an indicator variable for year and controls for maternal age, parity, race/ethnicity, education, and child gender.

^f^ Model includes birth records from 2003-2007.

^g^ Coefficients are estimated for singleton births between using logistic regression models, with county fixed-effects.
