# Supplementary material for: Unemployment during the Great Recession and Large-for-Gestational-Age births
Source: PLoS One. 2020 May 29;15(5):e0233734. doi: 10.1371/journal.pone.0233734 (PMC7259553; doi:10.1371/journal.pone.0233734)
Supplement: S2 Table — (DOCX) [file pone.0233734.s002.docx]

| **S2 Table. County Fixed-Effects Regression Estimates for the Relationship Between Unemployment Rate and Excess GWG, 2008-2011, Stratified by Race/Ethnicity** | | | |
| --- | --- | --- | --- |
|  | **n** | **Percentage Point**  **(95% CI)^a,b^** | **p value**^c^ |
| White | 432,562 | 0.27 (-0.28, 0.82) | 0.33 |
| African American or Black | 77,831 | 0.48 (-1.06, 2.03) | 0.53 |
| Hispanic or Latino | 816,057 | 0.04 (-0.51, 0.58) | 0.89 |
| Asian | 143,205 | 0.02 (-0.79, 0.82) | 0.97 |
| American Indian/Alaska Native | 4,594 | 0.89 (-3.69, 1.58) | 0.43 |
| Pacific Islander | 7,017 | -1.41 (-3.66, 0.83) | 0.21 |
| Filipino | 42,220 | 1.83 (0.46, 3.19) | 0.01 |
| Other | 977 | 1.93 (-2.94, 6.80) | 0.43 |
| Two or More Races | 67,496 | 0.20 (-0.34, 0.74) | 0.47 |

CI = confidence interval, GWG = gestational weight gain

^a^ Coefficients are estimated for singleton, term births between 2008-2011 using linear probability models, with county fixed-effects. All models include an indicator variable for year and control for county-level foreclosure rates, maternal age, parity, education, and child gender.

^b^ Beta coefficients are multiplied by 100 and can be interpreted as a percentage point change.

^c^ The *nonest* option was used to allow for non-nested county-level clustered standard errors.
