# Supplementary material for: Correction: Unemployment during the Great Recession and Large-for-Gestational-Age births
Source: PLoS One. 2020 Jul 29;15(7):e0236969. doi: 10.1371/journal.pone.0236969 (PMC7390381; doi:10.1371/journal.pone.0236969)
Supplement: S1 File — (PDF) [file pone.0236969.s001.pdf]

PLOS ONE

RESEARCH ARTICLE

Unemployment during the Great Recession and large-for-gestational age births

Vanessa M. Oddo[1,2]*, **Jessica C. Jones-Smith**[1,3]

1 Department of Health Services, University of Washington School of Public Health, Seattle, Washington, United States of America, **2** Department of Kinesiology and Nutrition, University of Illinois at Chicago, Chicago, Illinois, United States of America, **3** Department of Epidemiology, University of Washington School of Public Health, Seattle, Washington, United States of America

* voddo@uic.edu

Check for updates

OPEN ACCESS

Citation: Oddo VM, Jones-Smith JC (2020) Unemployment during the Great Recession and large-for-gestational age births. PLoS ONE 15(5): e0233734. https://doi.org/10.1371/journal.pone.0233734

Editor: JJ Cray, Jr., Ohio State University, UNITED STATES

Received: January 30, 2020

Accepted: May 11, 2020

Published: May 29, 2020

Copyright: © 2020 Oddo, Jones-Smith. This is an open access article distributed under the terms of the Creative Commons Attribution License, which permits unrestricted use, distribution, and reproduction in any medium, provided the original author and source are credited.

Data Availability Statement: We are legally prohibited from sharing this de-identified dataset. Data are owned by the California Department of Public Health's (CDPH)State Register. Per our data user agreement with the CDPH, only the first author, Dr. Vanessa Oddo and the University of Washington Manager of Departmental Computing are permitted access to the data files. Individuals wishing to use the California birth files must submit an application for access: California Department of Public Health Health information and Research HIRS@cdph.ca.gov https://www.cdph.ca.gov/

Abstract

Background

Several studies have suggested that record high unemployment during the Great Recession was associated with deleterious changes in diet and weight-related health. However, studies have yet to explore whether the Great Recession was also associated with obesity-related health *in utero*.

Methods

We investigated whether increasing county-level unemployment was associated with large-for-gestational age (LGA) births, using repeated cross-sectional data from California birth records between 2008 and 2011 (n = 1,715,052). LGA was defined as >90th percentile, using the Oken reference. We use the annual 1-year lagged value for county-level unemployment (2007–2010) and limit our analyses to singleton, term births. Linear probability models, with county and year fixed-effects were used to examine the unemployment-LGA association. All models control for county-level foreclosure rates, child gender, and maternal age, parity, education, and race/ethnicity.

Results

An increase in county-level unemployment was not statistically significantly associated with the prevalence of LGA (percentage point [PP]: 0.12; 95% CI: -0.02, 0.25). But, over the period of observation, for every one standard deviation increase in unemployment, LGA prevalence increased by 5% and p = 0.08.

Conclusions

These results cautiously suggest some deleterious effects of the Great Recession on obesity-related health *in utero*.

Programs/CHSI/Pages/Data-Applications.aspx.
916-440-7350

Funding: This work was supported by the National Institute on Minority Health and Health Disparities, Vanessa Oddo's salary was supported in part by the National Institute on Minority Health and Health Disparities (R00MD012807-03). The funders had no role in study design, data collection and analysis, decision to publish, or preparation of the manuscript.

Competing interests: The authors have declared that no competing interests exist.

Introduction

The recent Great Recession [1] was characterized by large increases in the unemployment rate and a slow recovery, as unemployment remained high into 2013 [2,3]. This persistent unemployment has been shown to be associated with deleterious changes in diet and weight-related health. Unemployment during the recession was associated with lower consumption of fruits and vegetables [4,5], higher consumption of energy-dense foods [6], increases in total calories purchased [7,8], higher body mass index [BMI] [9] and higher risk of child overweight [10] and adult obesity [9]. Studies have yet to explore whether unemployment during the Great Recession was also associated with obesity-related health *in utero*.

A relatively small body of literature has previously examined the association between individual-and aggregate-level unemployment and birthweight-related outcomes [11–19]. For example, Catalano and Serxner investigated the effects of a threatened job loss for state government employees, due to proposal of Proposition 13 in Sacramento, California. Using Proposition 13 as a marker for employment insecurity, the authors compared low birthweight (LBW) prior to the law passing (June 1978-February 1979), to LBW incidence after the law passed [18]. Employment insecurity was associated with a higher risk of LBW among males [18]. On the contrary, Dehejia and Lleras-Muney report that infants conceived during times of high national-level unemployment (1975–1999) had a lower prevalence of LBW [19]. More recently, Margerison-Zilko and colleagues investigated the association between state-level unemployment and preterm births (<37 weeks) between 2007 and 2009 [14]. The authors found that higher state-level unemployment, during the first trimester, was associated with 16% higher odds of preterm birth, whereas state-level unemployment during the second trimester was associated with 6% lower odds of preterm birth [14]. Although this small body of literature mostly suggests that aggregate-level unemployment is associated with adverse birth outcomes, some of these studies pre-dated the large increases in obesity prevalence [20] and changes in the food environment that occurred in the US during the 1990s [21], and all focused on undernutrition-related birth outcomes. We build upon this literature, and that which has explored diet and weight-related health during the Great Recession, by investigating obesity-related health *in utero*, as indicated by large-for-gestational-age (LGA) births.

There are multiple pathways through which recessions may affect birth outcomes [17]. The recession led directly to individual job loss and decreased individual-level income between 2009 and 2011 [22]. Recessions can induce "effect budgeting", which forces one to invest time into managing the sequelae of job and income loss, and subsequently, one stops investing in their well-being [23]. Work-related physical activity also declined during the Great Recession [24]. But recessions also impact people who do not actually lose their jobs, due to changes in social norms and a 'recession mentality' that occurs due to economic uncertainty. In addition, those who remain employed may have faced reduced hours or pay and/or experience stress due to fear of job loss [23,25]. High unemployment can also negatively impact communities and access to social services. Evidence links these intermediary factors to physiological changes in pregnant women that may affect gestation and subsequent birth outcomes. Primarily, high unemployment may increase the barriers around healthful eating and may result in decreased access to prenatal care and monitoring of gestational weight gain (GWG). Both pre-pregnancy BMI and GWG are associated with birthweight and length of gestation [26,27]. Relatedly, a recent systematic review reports associations between unhealthful dietary patterns and adverse birth outcomes (e.g. preterm births, LBW), although the authors did not find any studies that investigated unhealthy diets in relation to LGA [28]. In addition, recessions potentially change the population of women having babies; for example, if fewer lower-income women have children during economic downturns, due to additional economic constraints, this would change

the demographic distribution of infants [29]. Dehejia and Lleras-Muney show such selection during prior economic downturns in the US [29], and the US birth rate did decline between 2007 and 2014 [30,31]. Unplanned pregnancies may also be related both to recessions and heterogeneously distributed among the population studied [32,33].

A better understanding of the systemic risk factors for obesity-related health *in utero* are critical given the long-term implications of LGA on child obesity [34,35] and the unrelenting increases in obesity prevalence in the US. The primary aim of this paper was to investigate whether increasing unemployment during the Great Recession was associated with the likelihood of being born LGA. Our secondary aim was to test heterogeneity in the unemployment-LGA association by race/ethnicity and explore related outcomes: very-LGA, high birthweight, pre-pregnancy BMI, and excessive GWG.

Methods

Data sources

Maternal demographic and health characteristics, child sex, birthweight, and gestational age were obtained from the California birth records between 2008 and 2011. County-level annual unemployment estimates (2007–2010) were obtained from the Bureau of Labor Statistics. County-level annual foreclosure rates (2008–2011) were obtained from RealtyTrac LLC.

Dependent variables

The primary dependent variable was LGA births, defined as weight for gestational age $>90^{th}$ percentile compared with the 2000 U.S. birthweight reference [36]. Oken and colleagues provide the most recently published sex-and-gestational-age-specific birthweight reference for birthweight at 22 through 44 completed weeks [36]. We limited our primary analyses to singleton, term births, since reference charts may provide biased estimates of size among preterm births.

To address the secondary aims of this manuscript, we examined five other related dependent variables: LGA $>97^{th}$ percentile (termed "very-LGA"), high birthweight ($>$ 4000 grams) and preterm births (gestational age $<$ 37 weeks). We also investigated whether county-level unemployment was associated with maternal pre-pregnancy BMI (kg/m^2) and the probability of excessive GWG. We calculated pre-pregnancy BMI and GWG using self-reported maternal pre-pregnancy height and weight and measured delivery weight from the birth certificate. To calculate excessive GWG, we first subtracted maternal pre-pregnancy weight from maternal delivery weight. We then classified weight gain based on the Institute of Medicine guidelines for weight gain during pregnancy (underweight pre-pregnancy and gained $>$ 18 kg; normal weight and gained $>$15.9 kg; overweight and gained $>$ 11.4 kg; or, obese and gained more than 9 kg) [27].

Independent variables

Our main independent variable was one-year-lagged values of annual county-level unemployment (2007–2010). We use the value of county-level unemployment from the year preceding each newborn's birth (i.e. the one-year lagged value), since we would expect any impact from unemployment on birthweight to occur during or before pregnancy.

We explored the association between county-level unemployment (2007–2010) and LGA births (2008–2011) over a four-year period that overlaps with the Great Recession because the recession had longer-term economic impacts beyond its official end date in 2009 [22]. We examine aggregate, county-level unemployment (versus individual-level), because county-level

unemployment better captures changes in the macroeconomy and the “recession mentality”, and becoming unemployed is likely confounded with individual-level characteristics (e.g. work performance), that may also affect health [37]. Unemployment is also the most widely used indicator of recessions. Annual county-level unemployment rate (a percent) was defined as the number of persons unemployed divided by the civilian labor force, multiplied by 100.

Confounding factors, effect measure modifiers, and mediators

Time-invariant confounders (e.g. “baseline” county urbanization) were controlled for by using county fixed-effects in the regression models, which are explained below. We also include a year fixed-effect to control for secular decreases in LGA births in California over time.

Prior studies suggest that the composition of women having children changes during a recession [19]. Therefore, we control for maternal and child demographic and health characteristics. We also control for county-level foreclosure rates, which pre-dated the rise in unemployment rates and is a plausible confounder of the unemployment-LGA association. Annual county-level foreclosure rate (2008–2011) was defined as total foreclosures, divided by the total number of mortgages in the year of birth, multiplied by 100. Maternal and child characteristics included: maternal age (10–19, 20–29, 30–39, $\geq$ 40), parity (1, 2–5, 5–10, $>$10), maternal education ($\leq$ high school degree, $\geq$ some college, college graduate and above), maternal race/ethnicity and child gender. The California birth certificate included 21 race categories. Women self-reported up to three races, plus indicated if their ethnicity was Hispanic or Latino. In these analyses, race/ethnicity was aggregated as follows: Hispanic or Latino, White, African American or Black, Asian, American Indian/Alaska Native, Pacific Islander or Native Hawaiian, Filipino, Other, or Two or More races.

We hypothesized that the association between increasing unemployment and LGA births might be heterogenous by race/ethnicity [38]. Therefore, we tested whether race/ethnicity modified any association between unemployment and LGA births by including an interaction between race/ethnicity and unemployment. Smoking during pregnancy was hypothesized to be mediator of the association, so not controlled for in these analyses.

Statistical analysis

In our primary specification, we used linear probability models, with county and year fixed-effects, to test the association between changes in unemployment rates (2007–2010) and the probability of LGA births (2008–2011) in California. Standard errors were clustered at the county-level. By using county fixed-effects, we are able to compare each county to itself over time, and control for all baseline time-invariant measured and unmeasured confounding factors. Coefficients are probabilities and were multiplied by 100 so that they can be interpreted as the percentage point (PP) change in relation to a 1-pp increase in unemployment.

To address the secondary aims of this paper, we tested heterogeneity in the unemployment-LGA association by race/ethnicity with the inclusion of an interaction term (unemployment X race/ethnicity) and assessed statistical significance with a post-hoc Wald test of the interaction terms. The test of the interaction suggested that there was heterogeneity in the association by race/ethnicity (post-hoc Wald test $p < 0.01$); therefore, we also present race/ethnicity-stratified results.

Similar to our primary analyses, we used linear probability models, with county and year fixed-effects, to assess the relationship between unemployment and secondary outcomes: very-LGA, high birthweight, excessive GWG, and preterm births. Linear regression models, with county and year fixed-effects, were employed to assess the relationship between unemployment and pre-pregnancy BMI.

Sensitivity analyses

We assessed whether our primary results would have changed given the following alternative specifications: 1) when including preterm births; 2) when only including mothers that did not smoke during pregnancy; 3) excluding Los Angeles (LA) county, since 25% of Californians reside in LA; and 4) when excluding foreclosure rates as a covariate. We also assessed our primary results when excluding Pacific Islanders and people of an "Other" race, because post-hoc race/ethnicity-stratified results suggested that the magnitude of effect for these races were 30 times higher than the effect for people of other race/ethnicities. For comparison, we modeled the association between unemployment and LGA births during the years prior to the Great Recession (2003–2007). Finally, we employed logit regression models, and estimate average marginal effects, to test the association between unemployment and our primary and secondary outcomes, given that several outcomes (e.g. very LGA births) were rare.

Alpha was set to 0.05 and analyses were performed using Stata 15.1 (StataCorp LP, College Station, TX). The University of Washington School of Public Health deemed that this analysis of de-identified secondary data was not human subjects research. The California Health and Human Services Agency Committee for the Protection of Human Subjects approved the use of California birth records for these analyses.

Results

Over the observation period there were 138,970 LGA births in California (8.1% of all births) (Table 1). Statewide, unemployment was 9.2% between 2007 and 2010 (2007: 5.6 (standard deviation [SD] = 1.7); 2008: 7.5 (SD = 2.0); 2009: 11.5 (SD = 2.3); 2010: 12.5 (SD = 2.5). Based on 5-year estimates from the American Community Survey, the average county-level median household income in California was approximately $62,000 (SD = 12,137). The mean foreclosure rate was 7.3 (SD = 3.1). The mean age of mothers was 28.2 (standard deviation [SD] = 6.2) and a majority (75.8%) had at least some college education. More than half of the sample identified as Hispanic or Latino (51.8%) and one-quarter (26.8%) identified as White. Mean maternal pre-pregnancy BMI was 25.8 (SD = 5.9) and the prevalence of excessive GWG was approximately 47%.

Increasing county-level unemployment was not statistically significantly associated with prevalence of LGA, but the direction of the effect was positive and p = 0.08 (percentage point [PP]: 0.12; 95% Confidence Interval [CI]: -0.02, 0.25) (Table 2). Results were similar when employing logit models (marginal effect = 0.0011; 95% CI: -0.00019, 0.0023; p = 0.09) (S1 Table). Prior to the Great Recession (2003–2007) unemployment was not associated with LGA, but the direction of the association was negative, unlike our recession year results, and smaller in magnitude (PP = -0.05; 95% CI: -0.16, 0.07).

In race/ethnicity-stratified results, increasing unemployment was not associated with the prevalence of LGA for most race/ethnicities; however, among Filipinos, increasing county-level unemployment was associated with a significantly higher prevalence of LGA births (PP = 0.60; 95% CI: 0.22, 0.97) (Table 3). Although not statistically significant, the largest magnitude effects were observed among Pacific Islanders (PP = -1.74; 95% CI: -3.83, 0.35) and people of "Other" races (PP = 1.92; 95% CI: -2.82, 6.66).

Every 1-pp increase in unemployment was associated with a statistically significantly higher prevalence of very-LGA births (PP = 0.09; 95% CI: 0.03, 0.15) (Table 4). But, increasing unemployment was not associated with excess GWG (PP = 0.16; 95% CI: -0.35, 0.67), high birthweight (PP = 0.05; 95% CI: -0.09, 0.18), or pre-pregnancy BMI (PP = 0.03; 95% CI: -0.02, 0.08), although the direction of these effects was positive. On the contrary, unemployment during

Table 1. Key sample characteristics, 2008–2011[a].

Newborns, n	1,715,052
Economic Conditions	
County-Level Unemployment Rate, mean (sd)	9.2 (3.6)
2007	5.6 (1.7)
2008	7.5 (2.0)
2009	11.5 (2.3)
2010	12.5 (2.5)
County-Level Median Household Income, mean (sd)	61,728 (12,137)
County-Level Foreclosure Rate, mean (sd)	7.3 (3.1)
Child Characteristics and Health	
LGA Births, n (%)[b]	138,970 (8.1%)
Preterm Births, n (%)[c]	163,116 (8.7%)
Child Gender (Males), n (%)	873,268 (49.1%)
Maternal Characteristics and Health	
Maternal Age (years), mean (sd)	28.2 (6.2)
Maternal Education ($\geq$ Some College), n (%)	1,299,328 (75.8%)
Maternal Race/Ethnicity[d]	
Hispanic or Latino, n (%)	889,254 (51.8%)
White, n (%)	458,952 (26.8%)
African American or Black, n (%)	85,917 (5.0%)
Asian, n (%)	155,650 (9.1%)
American Indian/Alaska Native, n (%)	5,109 (0.30%)
Filipino, n (%)	44,680 (2.6%)
Pacific Islander, n (%)	7,017 (0.40%)
Other, n (%)	977 (0.10%)
Two or More, n (%)	67,496 (3.9%)
Pre-pregnancy Body Mass Index, mean (sd) [e]	25.8 (5.9)
Excessive gestational weight gain, n (%)[f]	743,777 (46.8%)
Smoking during pregnancy, n (%)	37,879 (2.2%)
Parity, mean (sd)	2.1 (1.2)

LGA = large-for-gestational-age; sd = standard deviation

[a] Descriptive statistics reflect the total observations over the time period (2008–2011).

[b] LGA births are defined as > 90th percentile compared with the Oken sex-and-gestational-age-specific reference population values.

[c] Preterm is defined as < 37 weeks gestation.

[d] Race/ethnicity was self-reported on the birth certificate.

[e] Maternal pre-pregnancy BMI was based on self-reported pre-pregnancy weight and height and calculated as weight (kg)/height^2 (m).

[f] Excessive weight gain was defined based on the Institute of Medicine definition: underweight (BMI < 18.5 kg/m^2) and gained more than 18 kg; normal weight ($\geq$ BMI 18.5 and < 25 kg/m^2) and gained more than 15.9 kg; overweight (BMI $\geq$ 25 and < 30 kg/m^2) and gained more than 11.4 kg; or obese (BMI $\geq$ 30 kg/m^2) and gained more than 9 kg.

https://doi.org/10.1371/journal.pone.0233734.t001

the recession was associated with a lower prevalence of preterm births (PP = -0.18; 95% CI: -0.31, -0.06).

Discussion

This study leveraged repeat cross-sectional data from birth certificates in California to identify whether increasing county-level unemployment, during the Great Recession, was associated

Table 2. County fixed-effects regression for the relationship between unemployment rate and LGA births in California, 2008–2011.

	n	Percentage Point (95% CI)[a]	p value[b]
Main Model			
Unemployment Rate[c,d]	1,715,052	0.12 (-0.02, 0.25)	0.08
Sensitivity Analyses			
Unemployment Rate, Including Preterm Births[c,d]	1,877,359	0.07 (-0.07, 0.20)	0.35
Unemployment Rate, Among Non-Smokers[c,d]	1,656,120	0.12 (-0.01, 0.25)	0.08
Unemployment Rate, Excluding LA County[c,d]	1,570,751	0.12 (-0.02, 0.26)	0.09
Unemployment Rate, Excluding Pacific Islanders and People of Other Races[c,e]	1,707,058	0.12 (-0.01, 0.25)	0.07
Unemployment Rate, Excluding Foreclosure[c,f]	1,715,052	0.10 (-0.03, 0.23)	0.14
Unemployment Rate, Pre-Recession Years[c,d,g]	2,145,755	-0.05 (-0.16, 0.07)	0.42

CI = confidence interval, LGA = large-for-gestational-age

[a] Beta coefficients were multiplied by 100 and can be interpreted as a percentage point change.

[b] The *nonest* option was used to allow for non-nested county-level clustered standard errors.

[c] Coefficients are estimated for singleton, term births between 2008–2011 using linear probability models, with county fixed-effects, to test the relationship between county-level unemployment and LGA births. County-level unemployment is lagged and reflects the unemployment rate in the year prior to birth.

[d] Models include an indicator variable for year and control for county-level foreclosure rates, maternal age, parity, maternal race/ethnicity, maternal education, child gender.

[e] Model includes an indicator variable for year and controls for county-level foreclosure rates, maternal age, parity, maternal education, child gender.

[f] Model includes an indicator variable for year and controls for maternal age, parity, maternal race/ethnicity, maternal education, child gender.

[g] Model includes birth records from 2003–2007.

https://doi.org/10.1371/journal.pone.0233734.t002

with LGA births. Country-level unemployment was not statistically significantly associated with LGA births, although the direction of the association was positive (0.12-pp) and the p-value was < 0.10. Moreover, over the period of observation, for every one standard deviation increase in unemployment, LGA prevalence increased by 0.43-pp or 5%. Although the estimate is not statistically significant at the 0.05 level, this is a meaningful effect. In addition, an

Table 3. County fixed-effects regression for the relationship between unemployment rate and LGA births, 2008–2011, stratified by race/ethnicity.

	N	Percentage Point (95% CI)[a,b]	p value[c]
Hispanic or Latino	889,254	0.10 (-0.07, 0.27)	0.24
White	458,952	0.06 (-0.16, 0.27)	0.60
African American or Black	85,917	0.24 (-0.20, 0.67)	0.29
Asian	155,650	0.22 (-0.12, 0.56)	0.19
American Indian/Alaska Native	5,109	0.66 (-1.03, 2.35)	0.41
Pacific Islander	7,017	-1.74 (-3.83, 0.35)	0.10
Filipino	44,680	0.60 (0.22, 0.97)	0.01
Other	977	1.92 (-2.82, 6.66)	0.43
Two or More Races	67,496	0.20 (-0.33, 0.74)	0.45

CI = confidence interval, LGA = large-for-gestational-age

[a] Coefficients are estimated for singleton, term births between 2008–2011 using linear probability models, with county fixed-effects, to test the relationship between lagged county-level unemployment to and LGA births. All models include an indicator variable for year and control for county-level foreclosure rates, maternal age, parity, maternal education, and child gender.

[b] Beta coefficients were multiplied by 100 and can be interpreted as a percentage point change.

[c] The *nonest* option was used to allow for non-nested county-level clustered standard errors.

https://doi.org/10.1371/journal.pone.0233734.t003

Table 4. County fixed-effects regression for the relationship between unemployment rate and secondary outcomes, 2008–2011.

	n	Percentage Point or β (95% CI)[a]	p value[b]
Very LGA[c]	1,715,052	0.09 (0.03, 0.15)	0.01
Excess GWG[c]	1,587,721	0.16 (-0.35, 0.67)	0.53
High Birthweight[c]	1,715,052	0.05 (-0.09, 0.18)	0.50
Pre-Pregnancy BMI[d]	1,601,815	0.03 (-0.02, 0.08)	0.26
Preterm[e]	1,878,201	-0.18 (-0.31, -0.06)	0.01

BMI = body mass index; CI = confidence interval; GWG = gestational weight gain; LGA = large-for-gestational-age

[a] All models include an indicator variable for year and control for county-level foreclosure rates, maternal age, maternal race ethnicity, parity, maternal education, and child gender.

[b] The *nonest* option was used to allow for non-nested county-level clustered standard errors.

[c] Coefficients are estimated for women who had singleton, term births between 2008–2011 using linear probability models, with county fixed-effects. Beta coefficients were multiplied by 100 and can be interpreted as a percentage point change.

[d] The coefficient is estimated for women who had singleton, term births between 2008–2011 using linear regression models, with county fixed-effects.

[e] The coefficient is estimated for women who had singleton births between 2008–2011 using linear probability models, with county fixed-effects. Beta coefficients were multiplied by 100 and can be interpreted as a percentage point change.

https://doi.org/10.1371/journal.pone.0233734.t004

increase in county-level unemployment was associated with a statistically significantly higher prevalence of very-LGA births. Our analysis of subgroups revealed some differences in the statistical significance and magnitude of effect for the unemployment-LGA association by race/ethnicity. Increasing county-level unemployment was associated with a higher prevalence of LGA births among Filipinos. An increase in county-level unemployment was also significantly associated with a lower prevalence of preterm births during the recession.

The direction of the unemployment-LGA association, and the fact that an increase in county-level unemployment was associated with a higher risk of very-LGA births is generally consistent with prior literature, which suggests adverse effects of the Great Recession on diet [4–8] and weight-related health [9,10]. The Great Recession has been shown to be associated with small increases in total calories purchases (1.6–4.1 kcal/capita/day) [7] and in the short-term, was associated with substitution in favor of discount stores and increases in consumption of fat [6]. During the recession, consumption of away from home foods continued [39] and there was a shift in spending away from higher-end, sit-down restaurants to cheaper options, like fast food [40]. Relatedly, we have previously shown that during the Great Recession, county-level unemployment was associated with higher overweight/obesity risk among school-age children in California [10] and Zhang and colleagues found that county-level unemployment rates were significantly associated with a higher BMI among adults in the US [9]. This prior literature on the Great Recession does generally support our finding that the direction of the association between increasing county-level unemployment and LGA was positive, and positive and statistically significant for increasing unemployment and very-LGA births. At the same time, effects of recessions on health are notably heterogenous. For example, despite several early studies (1970–1990) reporting that high or unexpected unemployment during recessions had adverse effects on birth outcomes, two additional studies report null associations for the relationship between aggregate unemployment and LBW in New York City [41] and in Tennessee [42] and other economic downturns have been associated with *decreased* overweight among adults [37,43].

Relatedly, our findings suggest the unemployment-LGA association is heterogenous by race/ethnicity, which is generally consistent with prior studies [38]. But, the fact that the race/ethnicity stratified association between county-level unemployment and LGA births was only statistically significant among Filipinos was unexpected. Notably, many Filipinos

resided in in LA county, which had higher than average unemployment (10.9% versus 9.2% overall) and county-level unemployment was associated with a significantly higher probability of excessive GWG among Filipinos (S2 Table). We speculate that larger excess GWG during pregnancy among Filipinos is driving the observed association in this population. We also observed large, although non-significant, changes in excessive GWG in relation to county-level unemployment for both Pacific Islanders (-1.4-pp) and people of an "Other" race (1.93-pp) that corresponded to the direction and magnitude of the association between unemployment and LGA. However, we are unsure of why the recession might disproportionally affect these populations.

Unexpectedly, increasing annual county-level unemployment was associated with a significantly lower prevalence of preterm births. Although these results were contrary to our hypothesis, Margerison-Zilko and Luo also report unexpected results. These authors find that during the recession (2007–2008), state-level unemployment in the first trimester of pregnancy was associated with higher odds of preterm births, whereas state-level unemployment during the second trimester of pregnancy was associated with lower odds of preterm birth [14]. Thus, the authors' findings are specific to the trimester of exposure and perhaps the defined recession period, as they define the recession as 2007–2009, when unemployment rates were still only modestly high in many states. They also use monthly state-level (versus annual county-level) unemployment and one prior study in the US does find contradictory results, within the same dataset, when using state- versus county-level unemployment as the exposure variable [9]. Notably, from 2008 to 2009, birth rates decreased by 5.9% among Hispanic women and 2.4% among Black women, compared to only 1.6% among White women [30] and Hispanic Black women tend to have higher rates of preterm births [44]. Also, from 2007 to 2014, preterm birth rate decreased from 10.4% to 9.5% nationally, which is attributed to a decline in teen pregnancy and shift in the age distribution of women giving birth [31]. Similarly, preterm births decreased from 10.6% to 9.8% between 2008 and 2011 in this sample. We do include a year fixed effect to control for secular trends and control for demographic characteristics, including race/ethnicity and age, but it is also possible that there is some residual confounding of the changes in the demographic of women having babies during the recession. It is also possible that these unexpected results are due to statistical chance.

Our data analysis and approach have several strengths including using a large sample of births and using county fixed-effects, which controls for time-fixed county-level unobservable factors. However, limitations should be noted. We are not able to control for potential unmeasured time-varying confounding; but biased coefficients would only result if a factor covaried with county-level unemployment and affected LGA. Relatedly, we do not have repeated birth records from the same women, which could allow us to better estimate causal effects and control for individual-level time-fixed unobservables (e.g. genetics). Gestational age is reliant on women's self-report of last menstrual period (LMP); although LMP is very commonly used in research to calculate gestational age, some studies suggest that up to 20% of women underreport the length of time since their LMP [45]. Findings may be less generalizable to states that experienced a less severe recession.

Conclusions

A better understanding of the systemic risk factors for LGA are critical given the long-term implications for health [34]. Increasing levels of unemployment was not significantly associated with LGA births, but the direction of the association was positive and increasing county-level unemployment was significantly associated with a higher prevalence of very-LGA births. This cautiously suggests some deleterious effects of the Great Recession on obesity-related

health *in utero*. Future studies could consider investigating the effects of the Great Recession on birth outcomes using longitudinal data, with repeated birth records.

Supporting information

S1 Table. Marginal effects for the relationship between unemployment rate and LGA, 2008–2011.
(DOCX)

S2 Table. County fixed-effects regression for the relationship between unemployment rate and excess GWG, 2008–2011, stratified by race/ethnicity.
(DOCX)

Author Contributions

Conceptualization: Vanessa M. Oddo, Jessica C. Jones-Smith.

Formal analysis: Vanessa M. Oddo.

Methodology: Vanessa M. Oddo, Jessica C. Jones-Smith.

Writing – original draft: Vanessa M. Oddo.

Writing – review & editing: Jessica C. Jones-Smith.

References

1. Bureau of Labor Statistics. Business Cycle Dating Committee [Internet]. 2010. http://www.nber.org/cycles/sept2010.html
2. National Conference of State Legislators. National Employment Monthly Update [Internet]. 2019. http://www.ncsl.org/research/labor-and-employment/national-employment-monthly-update.aspx
3. Borbely JM. Sizing up the 2007–09 recession: comparing two key labor market indicators with earlier downturns. Issues Labor Stat. US Bureau of Labor Statistics; 2010; 10–11.
4. Bartoll X, Toffolutti V, Malmusi D, Palència L, Borrell C, Suhrcke M. Health and health behaviours before and during the Great Recession, overall and by socioeconomic status, using data from four repeated cross-sectional health surveys in Spain (2001–2012). BMC Public Health. 2015; 15: 865.
5. Dave DM, Kelly IR. How does the business cycle affect eating habits? Soc Sci Med. 2012; 74: 254–262.
6. Smed S, Tetens I, Lund TB, Holm L, Nielsen AL. The consequences of unemployment on diet composition and purchase behaviour: a longitudinal study from Denmark. Public Health Nutr. 2018; 21: 580–592.
7. Ng SW, Slining MM, Popkin BM. Turning point for US diets? Recessionary effects or behavioral shifts in foods purchased and consumed. Am J Clin Nutr. 2014; 99: 609–616.
8. Griffith R, O'Connell M, Smith K. Shopping around? How households adjusted food spending over the Great Recession. Economica. CEPR Discussion Paper No. DP10096; 2016; 83: 247–280.
9. Zhang Q, Lamichhane R, Wang Y. Associations between US adult obesity and state and county economic conditions in the recession. J Clin Med. 2014; 3: 153–166.
10. Oddo VM, Nicholas LH, Bleich SN, Jones-Smith JC. The impact of changing economic conditions on overweight risk among children in California from 2008 to 2012. J Epidemiol Community Heal. 2016; jech-2015-207117.
11. Rodrigues T, Barros H. Maternal unemployment: an indicator of spontaneous preterm delivery risk. Eur J Epidemiol. 2008; 23: 689.
12. Scharber H. Does "out of work" get into the womb? Exploring the relationship between unemployment and adverse birth outcomes. J Health Soc Behav. 2014; 55: 266–282.
13. Dooley D, Prause J. Birth weight and mothers' adverse employment change. J Health Soc Behav. 2005; 46: 141–155.
14. Margerison-Zilko CE, Li Y, Luo Z. Economic conditions during pregnancy and adverse birth outcomes among singleton live births in the United States, 1990–2013. Am J Epidemiol. 2017; 186: 1131–1139.

15. Fisher ES, LoGerfo JP, Daling JR. Prenatal care and pregnancy outcomes during the recession: the Washington State experience. Am J Public Health. 1985; 75: 866–869.
16. Catalano R, Hansen H-T, Hartig T. The ecological effect of unemployment on the incidence of very low birthweight in Norway and Sweden. J Health Soc Behav. 1999; 422–428.
17. Margerison Zilko CE. Economic contraction and birth outcomes: an integrative review. Hum Reprod Update. 2010; 16: 445–458.
18. Catalano R, Serxner S. The effect of ambient threats to employment on low birthweight. J Health Soc Behav. 1992; 363–377.
19. Dehejia R, Lleras-Muney A. The Timing of Births: Is the Health of Infants Counter-Cyclical? National Bureau of Economic Research; 2003.
20. Flegal KM, Carroll MD, Kuczmarski RJ, Johnson CL. Overweight and obesity in the United States: prevalence and trends, 1960–1994. Int J Obes. 1998; 22: 39.
21. James P, Seward MW, O'Malley AJ, Subramanian SV, Block JP. Changes in the food environment over time: examining 40 years of data in the Framingham Heart Study. Int J Behav Nutr Phys Act. 2017; 14: 84.
22. Green G, Coder J. Household Income Trends During the Recession and Economic Recovery [Internet]. 2011. http://www.sentierresearch.com/pressreleases/SentierResearch_PressRelease_October_10_2011.pdf
23. Catalano R, Goldman-Mellor S, Saxton K, Margerison-Zilko C, Subbaraman M, LeWinn K, et al. The health effects of economic decline. Annu Rev Public Health. 2011; 32: 431–450. https://doi.org/10.1146/annurev-publhealth-031210-101146 PMID: 21054175
24. Colman G, Dave D. Exercise, physical activity, and exertion over the business cycle. Soc Sci Med. 2013; 93: 11–20.
25. Kasl SV, Gore S, Cobb S. The experience of losing a job: reported changes in health, symptoms and illness behavior. Psychosom Med. 1975;
26. Pawlak MT, Alvarez BT, Jones DM, Lezotte DC. The effect of race/ethnicity on gestational weight gain. J Immigr Minor Heal. 2015; 17: 325–332.
27. Institute of Medicine. Influence of Pregnancy Weight on Maternal and Child Health Workshop Report. Washington, DC; 2007.
28. Chia A-R, Chen L-W, Lai JS, Wong CH, Neelakantan N, van Dam RM, et al. Maternal Dietary Patterns and Birth Outcomes: A Systematic Review and Meta-Analysis. Adv Nutr. 2019;
29. Dehejia RH, Lleras-Muney A. Booms, busts, and babies' health. National Bureau of Economics Research; 2004.
30. Livingston G, Cohn D. US birth rate decline linked to recession. Pew Soc Demogr Trends. 2010; [Internet]. 2019. https://www.pewsocialtrends.org/2010/04/06/us-birth-rate-decline-linked-to-recession/.
31. Ferré C. Effects of maternal age and age-specific preterm birth rates on overall preterm birth rates—United States, 2007 and 2014. MMWR Morb Mortal Wkly Rep. 2016; 65.
32. Finer LB, Zolna MR. Declines in unintended pregnancy in the United States, 2008–2011. N Engl J Med. 2016; 374: 843–852.
33. Gold R, Nash E, Cohen SA, Boonstra H, Saul R, Dailard C, et al. A real-time look at the impact of the recession on women's family planning and pregnancy decisions. New York Guttmacher Inst. 2009;
34. Mehta SH, Kruger M, Sokol RJ. Being too large for gestational age precedes childhood obesity in African Americans. Am J Obstet Gynecol. 2011; 204: 265. e1–265. e5.
35. Geserick M, Vogel M, Gausche R, Lipek T, Spielau U, Keller E, et al. Acceleration of BMI in early childhood and risk of sustained obesity. N Engl J Med. 2018.
36. Oken E, Kleinman KP, Rich-Edwards J, Gillman MW. A nearly continuous measure of birth weight for gestational age using a United States national reference. BMC Pediatr. 2003; 3: 6.
37. Ruhm CJ. Are recessions good for your health? Q J Econ. 2000; 115: 517–650.
38. Currie J, Duque V, Garfinkel I. The Great Recession and Mothers' Health. Econ J. 2015; 125: F311–F346.
39. Smith LP, Ng SW, Popkin BM. Resistant to the Recession: Low-Income Adults' Maintenance of Cooking and Away-From-Home Eating Behaviors During Times of Economic Turbulence. Am J Public Health. 2014; 104: 840–846.
40. Beatty TKM, Senauer B. The new normal? US food expenditure patterns and the changing structure of food retailing. Am J Agric Econ. 2012; 95: 318–324.
41. Joyce T. A time-series analysis of unemployment and health: The case of birth outcomes in New York city. J Health Econ. 1990; 8: 419–436.

42. Joyce TJ, Mocan NH. Unemployment and Infant Health: Times-Series Evidence from the State of Tennessee. National Bureau of Economic Research; 1991.

43. Ruhm CJ. Healthy living in hard times. J Health Econ. Elsevier; 2005; 24: 341–363.

44. Martin JA, Hamilton BE, Osterman MJK, Driscoll AK, Drake P. Births: final data for 2016. Natl Vital Stat Rep. 2018; 67(1):1–5.

45. Wegienka G, Baird DD. A comparison of recalled date of last menstrual period with prospectively recorded dates. J Women's Heal. 2005; 14: 248–252.
